# Guidance of children visiting in the adult intensive care by nurses

**DOI:** 10.1186/2197-425X-3-S1-A724

**Published:** 2015-10-01

**Authors:** EM de Vries-van Hooff, C de Kuijer, T van Galen

**Affiliations:** RN, VU University Medical Center, Intensive Care, Amsterdam, Netherlands; Holland University, Amsterdam, Netherlands; ICU Nursing, VU University Medical Center, Intensive Care, Amsterdam, Netherlands

## Introduction

At the 22 bed ICU at VU University medical center (VUmc) there was no uniform approach to the guidance offered by nurses to children visiting the adult ICU. Visitation was frequently discouraged. No educational material was available to prepare a child for a visit. The guidance of children does not form part of ICU training. Research has shown it is good to allow children on the adult ICU provided that they are well prepared and guided (Vint, 2005)[1]. These research findings together with observations during day-to-day practice were reason for further study and applying interventions for improvement. The target group is children between the ages of 4 to 12.

## Methods

A literature study was performed, followed by a survey with open and closed questions for nurses working in the adult ICU. Interventions were developed and implemented by a multidisciplinary focus group consisting of an IC nurse, a bachelor student and pedagogical colleagues.

## Results

85 nurses received the survey. The response rate was 80%. The survey results confirmed the observation made during day-to-day practice. 10% of the respondents indicated that a uniform approach was used by nurses to provide guidance.

The survey showed that the guidance provided was based mainly on intuition.

The focus group initiated the following interventions:

1. Protocol for the guidance of children by nurses to promote a uniform approach.
2. An informative picture book explaining what to expect while visiting the ICU, written in a language understood by children. This book can be read to by family or a nurse or by the child itself from ages 7 and up.
3. A backpack is available for children who are in need of more information after a visit. The backpack contains materials used in the ward. This will help the children to make it palpable.

## Discussion

After a short research period three interventions were implemented to improve children's guidance offered by the ICU nurses before and during a visit. In the past visits to the ICU by children were frequently discouraged, now visits are encouraged and the educational materials are leading to a more uniform approach to providing guidance. In addition to the above interventions parents are provided with an informative leaflet to help prepare a child for a visit. The picture book is part of the ICU website. In September 2015 the results of the interventions will be measured among the IC nurses. On January 30^th^ 2015 a national workgroup was formed: **F**amily and patient **C**entered **I**ntensive **C**are[2] (FCIC), with the aim of achieving national alignment on this topic.

## References

[1] Vint P (2005). Children visiting adults in ITU. Nursing in Critical Care.

[2] [http://www.opeenicliggen.nl/family-centered-intensive-care-fcic/]

